# High Update Rate Attitude Measurement Method of Star Sensors Based on Star Point Correction of Rolling Shutter Exposure

**DOI:** 10.3390/s21175724

**Published:** 2021-08-25

**Authors:** Longdong He, Yuebo Ma, Rujin Zhao, Yaxian Hou, Zifa Zhu

**Affiliations:** 1 Institute of Optics and Electronics of the Chinese Academy of Sciences, Chengdu 610209, China; helongdong18@mails.ucas.ac.cn (L.H.); mayuebo@ioe.ac.cn (Y.M.); houyaxian17@mails.ucas.ac.cn (Y.H.); zhuzifa18@mails.ucas.ac.cn (Z.Z.); 2 University of Chinese Academy of Sciences, Beijing 100049, China; 3 Key Laboratory of Science and Technology on Space Optoelectronic Precision Measurement, Chinese Academy of Sciences, Chengdu 610209, China

**Keywords:** start sensor, rolling shutter exposure, star point correction, attitude update, recursive estimation

## Abstract

Attitude update rate is one of the important indicators of star sensor performance. In order to resolve the problem of the low attitude update rate of star sensors, this paper proposes a star sensor attitude update method based on star point correction of rolling shutter exposure. Based on the characteristics of the asynchronous exposure of the rolling shutter, recursive estimation of the motion attitude and the corrected star point information were combined to realize multiple updates of the attitude in a single frame of the star map. Simulation and experimental results proved that the proposed method could increase the attitude update rate of a star sensor by 15 times, up to 150 Hz.

## 1. Introduction

The star sensor is the most accurate sensor for spacecraft attitude measurement [1]. It is a device that observes stars in different positions and provides accurate attitude information for the spacecraft [2]. Currently, the attitude update rate of the star sensor is less than 20 Hz. With the rapid development of space tasks, including high-resolution Earth observations and high-precision surveying and mapping, the problem of low update rates has become an important bottleneck restricting the progress of the aerospace field, especially remote sensing space technology [3]. Improvement of the attitude update rate can effectively solve the problem of attitude continuity, improving the rapid maneuverability and stability of the satellite platform.

Since star energy is weak, the longer exposure time is a major challenge for star sensors to improve the attitude update rate. A star sensor mainly has two exposure methods: global exposure and rolling shutter exposure [4]. Global exposure adopts the entire frame of the image exposure and reads it row by row. Each frame of the image comprises a posture measurement, and the posture update rate is constrained by the exposure time and the readout time. Rolling shutter exposure is a line-by-line exposure. Both the exposure and the reading are performed simultaneously. Rolling shutter exposure has a fast imaging speed [5], and the image also contains the posture information of the moving object during the exposure time, which has the potential to improve the posture update rate.

Various research studies have been conducted to improve the attitude update rate of star sensors, mainly from the twin aspects of hardware and algorithms. In terms of hardware, Wenbo Yu et al. [6] proposed an enhanced multiple exposure imaging method for star trackers. They recorded N groups of different star positions to form the star image and then obtained N groups of the corresponding attitude information, that is, the attitude update rate was increased by N times. However, the image intensifier increased the volume and quality of the star tracker and, at the same time, increased the power consumption. Xinpeng Li et al. [7] proposed a distributed field of view fusion method based on asynchronous exposure, using a multi-probe star sensor method that outputted the attitude of each probe to increase the attitude update rate. However, the multi-probe star sensor is bulky. Hongjun Zhong et al. [8] proposed a method based on pipeline parallel processing to improve the data update rate of a star sensor. In terms of algorithms, these are mainly based on the characteristics of rolling shutter exposure. Shuo Zhang et al. [9] proposed a attitude estimation method with a high update rate for star sensors in rolling exposure mode based on single-star point attitude recursive estimation, combined with an extended Kalman filter that realized one star per update point and estimated the posture once. However, this method has a single form of attitude update rate that cannot meet the requirements of a diversified attitude update rate in space missions. Hyosang Yoon [10] proposed a method to correct the image of the rolling shutter, which could solve the attitude error caused by the rolling shutter exposure, but the premise on which this method can be used is being able to identify the stars before calibration. When the star sensor moves fast, the star point position offset caused by the rolling shutter is large, which makes the sensor unable to complete the star map identification.

This study proposes a star sensor attitude measurement algorithm with a high update rate based on rolling shutter exposure star point correction. In the proposed method, the characteristics of asynchronous rolling shutter exposure are utilized to fuse the motion attitude recursive estimation with the corrected star point vector information in order to achieve multiple attitude updates in a single frame of the star map and improve the attitude update rate of the star sensor. The proposed method can flexibly select the attitude update rate based on the number of star points to meet the requirements of attitude update rate in different space missions.

## 2. Star Sensor Working Principle

The star sensor is a key device for determining the attitude of a spacecraft in orbit [11]. The workflow includes centroid extraction, star map recognition and attitude calculation [12]. When the star sensor is in orbit, the star is imaged through the optical system, and the centroid algorithm [13], the star pattern recognition algorithm [14] and the attitude estimation algorithm [15] are used to calculate the attitude of the spacecraft.

### 2.1. Imaging Model

**Figure 1:**
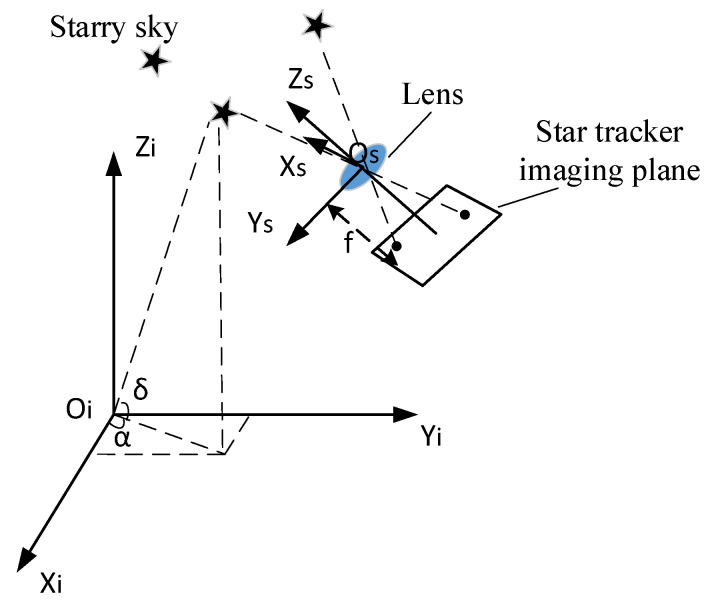
Star tracker imaging principle.

Figure 1 shows the imaging principle of a star sensor. The coordinates of the navigation star i in the celestial coordinate system are denoted as $\left(\alpha_{i},\delta_{i}\right)$ and represented by the right ascension α and the declination δ. According to the conversion relationship between the spherical and the rectangular coordinate systems [16], the direction vectors $v_{i}$ and $w_{i}$ can be written in the celestial Cartesian and the star sensor coordinate systems, respectively, as:

(1) $$v_{i}=\left[\begin{array}{c} \cos\alpha_{i}\cos\delta_{i}\\ \cos\alpha_{i}\sin\delta_{i}\\ \sin\delta_{i} \end{array}\right]$$

(2) $$w_{i}=\frac{1}{\sqrt{{\left(x_{i}-x_{0}\right)}^{2}+{\left(y_{i}-y_{0}\right)}^{2}+f^{2}}}\left[\begin{array}{c} -\left(x_{i}-x_{0}\right)\\ -\left(y_{i}-y_{0}\right)\\ f \end{array}\right]$$

where, $\left(x_{i},y_{i}\right)$ is the imaging position of the navigation star.

The algorithm must then satisfy:

(3) $$w_{i}=Av_{i}$$

where A is the attitude matrix of the star sensor.

The number of navigation stars involved in calculating the attitude of the star sensor is usually 5~15 [17]. The QUEST algorithm can be used to obtain the optimal estimation of the attitude by minimizing the value of the objective function [18,19]:

(4) $$J(A)=\frac{1}{2}\sum_{i=1}^{n}a_{i}||w_{i}-A_{q}v_{i}|{|}^{2}$$

where $a_{i}$ represents the weight coefficient of the navigation star point.

### 2.2. Rolling Shutter Exposure Mode

**Figure 2:**
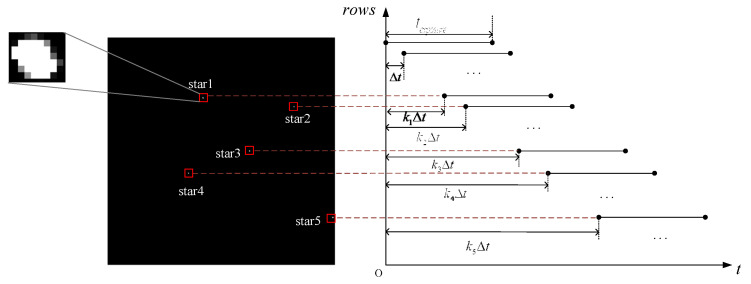
Rolling shutter exposure star diagram and imaging timing diagram.

Rolling shutter exposure mode is an exposure mode in which the CMOS image sensor performs line-by-line exposure with a delay between lines [20]. When the star sensor is working in the orbit, the position of the navigation star point in the imaging star map is shifted [21] and the shape is distorted due to the relative speed between the navigation star point and the satellite platform, as shown in Figure 2. If we define the number of coordinate rows of the *i*-th star point as $k_{i}$, and the corresponding delay time from the first line as $k_{i}\Delta t$, this distortion will seriously affect the star sensor’s star point positioning accuracy and attitude measurement accuracy.

## 3. High-Update-Rate Attitude Measurement Methods

Longdong He et al. [22] proposed a star point centroid correction method based on time-domain constraints to solve the problem of star point distortion and imaging position shift caused by rolling shutter exposure. By correcting the star point at an asynchronous time to the same time, the correction amount is:

(5) $$\left[\begin{array}{c} \Delta x\\ \Delta y \end{array}\right]=\left[\begin{array}{c} \left(y-k\right)v_{x}\Delta t\\ \left(y-k\right)v_{y}\Delta t \end{array}\right]$$

where x and y represent the coordinates of the star point before correction; $v_{x}$ and $v_{y}$ represent the speeds of the star point in the x and y directions, respectively; k represents the specified correction line and Δt is the line reset and read time.

The corrected star point coordinates are:

(6) $$\left[\begin{array}{c} x_{C}\\ y_{C} \end{array}\right]=\left[\begin{array}{c} x\\ y \end{array}\right]+\left[\begin{array}{c} \Delta x\\ \Delta y \end{array}\right]$$

After the navigation star points in different time domains in the star map have been corrected to the same time, the problem of star point distortion and position offset caused by the asynchronous exposure of the rolling shutter is effectively solved. Hence, the positioning accuracy of the navigation star point coordinates is improved.

The attitude calculation method based on Quest [23] is suitable for global exposure star sensor attitude measurement. All the star points start and end exposure at the same time. After calculation of the attitude information of a single moment has been completed, the previous star points’ measurement information will be abandoned. The attitude update rate of the star sensor is significantly affected by the exposure and readout time. For example, when the exposure and readout time is set to 100 ms, the attitude update rate of the star sensor is less than 10 Hz. The imaging of the navigation star point at different times of the rolling shutter exposure records the movement information of the star sensor during the exposure time, which is beneficial for improving the attitude update rate. The request-based attitude determination algorithm has theoretically proved that adding the star points’ measurement vector information at a single moment can realize the determination of the star sensor’s attitude information at that moment [24], and the star point correction method ensures that the star point is corrected at the same time. Hence, the positioning accuracy of the star point can effectively be improved.

**Figure 3:**
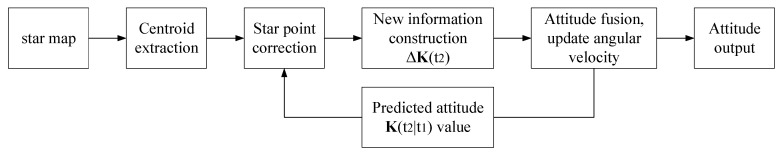
Block diagram of the high-update-rate attitude measurement algorithm.

Therefore, a high-update-rate attitude measurement algorithm for star sensors based on star point correction is proposed in this study. The proposed algorithm combines the continuous motion attitude recursive estimation and the corrected star point measurement information to obtain attitude information at different moments in a single frame of the star map and update the attitude multiple times during the exposure time. The proposed algorithm makes up for the problem of no posture information within the exposure time under the traditional global exposure mode. A block diagram of the proposed high-update-rate attitude measurement algorithm is shown in Figure 3. The characteristic matrix obtained by recursive estimation is defined as $K\left(t_{2}|t_{1}\right)$ and the characteristic matrix constructed by the new star point information after correction is $\Delta K\left(t_{2}\right)$.

Recursive estimation of the continuous motion attitude of the star sensor can be obtained using the satellite orbit attitude dynamics and control method proposed in [25]. From the attitude prediction at $t_{1}$ to $t_{2}$, the predicted $K\left(t_{2}|t_{1}\right)$ matrix at the time $t_{2}$ [26] can be obtained, which contains the components involved in calculation of the star point’s attitude-related information. The navigation star point during $t_{1}$~$t_{2}$ in the rolling star map is then corrected to time $t_{2}$, and the $\Delta K\left(t_{2}\right)$ matrix at $t_{2}$ is constructed based on the measurement information of the navigation star corrected to time $t_{2}$. The recursive $K\left(t_{2}|t_{1}\right)$ and $\Delta K\left(t_{2}\right)$ are merged to obtain the real $K\left(t_{2}|t_{2}\right)$ matrix that can be solved to obtain the posture information at the time $t_{2}$. The process is as follows:

According to [27], Formula (4) for finding the minimum value of the attitude solution objective function J(A) can be written as:

(7) $$J(A)=\frac{1}{2}\sum_{i=1}^{n}a_{i}||w_{i}-Av_{i}|{|}^{2}=1-q^{T}Kq$$

where q is the four-q element of the attitude, and K is the feature matrix constructed from the star point information.

Function g(q) is defined as:

(8) $$g(q)=q^{T}Kq$$

According to the related knowledge of attitude dynamics, the relationship between the quaternion difference equation and the angular rate can be obtained as [10]:

(9) $$\overset{•}{q}=\frac{1}{2}\Omega \left(\omega \right)q$$

where:

(10) $$\Omega \left(\omega \right)=[\begin{array}{cc} -[\omega \times ] & \omega \\ -\omega^{T} & 0 \end{array}]$$

Solving Equation (9) can provide the attitude prediction at time $t_{1}$ to $t_{2}$ [28]:

(11) $$q\left(t_{2}|t_{1}\right)=\Phi [t_{2},\omega \left(t_{1}\right)]q\left(t_{1}|t_{1}\right)$$

where:

(12) $$\Phi \left[t_{2},\omega \left(t_{1}\right)\right]=\cos\left(\frac{1}{2}||\omega \left(t_{1}\right)||\left(t_{2}-t_{1}\right)\right)I_{4}+\left[\begin{array}{cc} -\left[\Psi \left(t_{2},t_{1}\right)\times \right] & \Psi \left(t_{2},t_{1}\right)\\ -\Psi {\left(t_{2},t_{1}\right)}^{T} & 0 \end{array}\right]$$

and $\Psi \left(t_{2},t_{1}\right)$:

(13) $$\Psi \left(t_{2},t_{1}\right)=\frac{\sin\left(||\omega \left(t_{1}\right)||\left(t_{2}-t_{1}\right)/2\right)}{\left||\omega \left(t_{1}\right)|\right|}\omega \left(t_{1}\right)$$

where $\Phi \left[t_{2},\omega \left(t_{1}\right)\right]$ is the state transition matrix from $t_{1}$ to $t_{2}$, and is an oblique symmetric matrix that can be obtained using Formula (11):

(14) $$q\left(t_{1}|t_{1}\right)=\Phi^{-1}[t_{2},\omega \left(t_{1}\right)]q\left(t_{2}|t_{1}\right)=\Phi^{T}[t_{2},\omega \left(t_{1}\right)]q\left(t_{2}|t_{1}\right)$$

By substituting Equation (14) into Equation (8), the following can be obtained:

(15) $$g\left(q\left(t_{1}|t_{1}\right)\right)=q^{T}\left(t_{2}|t_{1}\right)\Phi [t_{2},\omega \left(t_{1}\right)]K\left(t_{1}|t_{1}\right)\Phi^{T}[t_{2},\omega \left(t_{1}\right)]q\left(t_{2}|t_{1}\right)$$

Let:

(16) $$K\left(t_{2}|t_{1}\right)=\Phi [t_{2},\omega \left(t_{1}\right)]K\left(t_{1}|t_{1}\right)\Phi^{T}[t_{2},\omega \left(t_{1}\right)]$$

We then define:

(17) $$g’\left(q\left(t_{2}|t_{1}\right)\right)=q^{T}\left(t_{2}|t_{1}\right)K\left(t_{2}|t_{1}\right)q\left(t_{2}|t_{1}\right)$$

By solving the maximum eigenvalue of Equation (17) and its corresponding eigenvector, the estimated posture from $t_{1}$ to $t_{2}$ can be obtained based on the angular velocity.

In order to obtain the true optimal posture at time $t_{2}$, the star point information at the time $t_{1}\sim t_{2}$ calculated from $t_{1}$ is added to the $K\left(t_{2}|t_{1}\right)$ matrix at time $t_{2}$. However, the rolling shutter exposure causes the exposure time of each star point to be different. Thus, according to the distribution of the star points in the star map, the star points in the time period $t_{1}\sim t_{2}$ need to be corrected. Therefore, the star points exposed in the time period $t_{1}\sim t_{2}$ are all corrected to the time $t_{2}$ using the start point correction method, and the measurement information at the time $t_{2}$ is obtained to form $\Delta K\left(t_{2}\right)$. The attitude information of the star sensor at $t_{2}$ is determined by combining the recursive $K\left(t_{2}|t_{1}\right)$ matrix and the $\Delta K\left(t_{2}\right)$ matrix constructed by the corrected measurement vector information.

**Figure 4:**
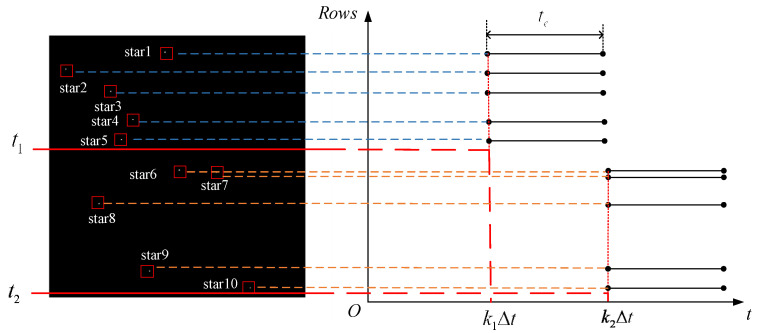
Rolling shutter exposure star point correction diagram.

Figure 4 shows a diagram of the rolling shutter exposure star point correction method. Suppose there are n star points involved in the attitude calculation at $t_{1}$, and the characteristic matrix at $t_{2}$ is estimated at $t_{1}$ through the angular velocity relationship. The coordinate measurement values of the m navigation star points exposed during the time $t_{1}\sim t_{2}$ are now corrected to time $t_{2}$ (based on the k_2_ row) and $\Delta K\left(t_{2}\right)$ is constructed according to the corrected information, which is combined with $K\left(t_{2}|t_{1}\right)$ to obtain the posture $K\left(t_{2}|t_{2}\right)$ at the time $t_{2}$.

According to the information of n corrected star points exposed before the time $t_{1}$ of the frame, define:

(18) $$b_{t_{1}}=\sum_{i=1}^{n}a_{i}$$

(19) $$\sigma =\frac{1}{b_{t_{1}}}\sum_{i=1}^{n}a_{i}w_{i}^{T}v_{i}$$

(20) $$B=\frac{1}{b_{t_{1}}}\sum_{i=1}^{n}a_{i}w_{i}v_{i}^{T}$$

(21) $$S=B+B^{T}$$

(22) $$z=\frac{1}{b_{t_{1}}}\sum_{i=1}^{n}a_{i}\left(w_{i}\times v_{i}\right)$$

Thus, K is:

(23) $$K=\left[\begin{array}{cc} S-\sigma I & z\\ z^{T} & \sigma \end{array}\right]$$

where I is a 3 × 3 identity matrix.

The coordinate measurement values of the m navigation star points exposed during the time $t_{1}\sim t_{2}$ are corrected to the information at $t_{2}$:

(24) $$\Delta b_{t_{1}\sim t_{2}}=\sum_{i=n+1}^{n+m}a_{i}$$

(25) $$\Delta \sigma_{t_{1}\sim t_{2}}=\sum_{i=n+1}^{n+m}a_{i}w_{i}^{T}v$$

(26) $$\Delta B_{{}_{t_{1}\sim t_{2}}}=\sum_{i=n+1}^{n+m}a_{i}w_{i}v_{i}^{T}$$

(27) $$\Delta S_{t_{1}\sim t_{2}}=B_{t_{1}\sim t_{2}}+B_{t_{1}\sim t_{2}}^{T}$$

(28) $$\Delta z_{t_{1}\sim t_{2}}=\sum_{i=n+1}^{n+m}a_{i}\left(w_{i}\times v_{i}\right)$$

The corrected measurement value is then used as the characteristic matrix constructed incrementally:

(29) $$\Delta K\left(t_{2}\right)=\left[\begin{array}{cc} \Delta S_{t_{1}\sim t_{2}}-\Delta \sigma_{t_{1}\sim t_{2}}I & \Delta z_{t_{1}\sim t_{2}}\\ \Delta z_{t_{1}\sim t_{2}}{}^{T} & \Delta \sigma_{t_{1}\sim t_{2}} \end{array}\right]$$

where:

(30) $$b_{t_{2}}=b_{t_{1}}+\Delta b_{t_{1}\sim t_{2}}$$

The $K\left(t_{2}|t_{2}\right)$ matrix after fusion with the recursive $K\left(t_{2}|t_{1}\right)$ is:

(31) $$K\left(t_{2}|t_{2}\right)=\frac{b_{t_{1}}}{b_{t_{2}}}K\left(t_{2}|t_{1}\right)+\frac{1}{b_{t_{2}}}\Delta K\left(t_{2}\right)$$

The optimal attitude quaternion $q^{*}\left(t_{2}|t_{2}\right)$ of the star sensor at time $t_{2}$ is obtained by calculating the maximum eigenvalue of $K\left(t_{2}|t_{2}\right)$ and its corresponding eigenvector as:

(32) $$K\left(t_{2}|t_{2}\right)q^{*}\left(t_{2}|t_{2}\right)=\lambda q^{*}\left(t_{2}|t_{2}\right)$$

The algorithm updates the angular velocity at the time $t_{2}$, and recursively estimates the $K\left(t_{2}|t_{2}\right)$ matrix to the next time.

According to the position distribution of the star points in each frame of the rolling exposure star map, updates of the attitude at different times in the rolling exposure star map can be realized using the request-based attitude measurement method combined with the star point correction method. Based on the number and the distribution of star points in rolling shutter exposure imaging, the algorithm is further expanded.

**Figure 5:**
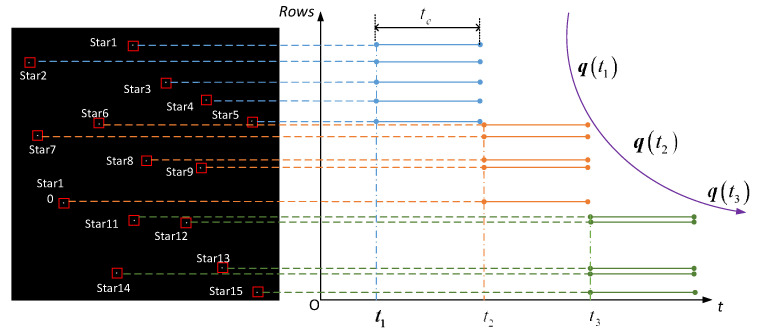
Schematic diagram of updating the attitude every five star points in a single-frame star map.

Generally, there are 5 to 15 star points in a single-frame star image of the star sensor. Now take 15 stars as an example. Figure 5 shows the process of updating the attitude of star points in a single frame star map, where 15 stars are evenly distributed from top to bottom in the star chart and each star point represents a different moment. Taking $t_{0}$ as the starting time of the first row, from top to bottom, every five stars can be taken to determine the attitude, and the rows of the 5th, 10th and 15th stars are used as the reference rows for calibration, that is, Stars 1~4 are calibrated to the exposure time of the fifth star, and the corrected innovation $\Delta K\left(t_{1}\right)$ is fused with the recursive $K\left(t_{1}|t_{0}\right)$ matrix to obtain $K\left(t_{1}|t_{1}\right)$. Hence, the attitude information at the exposure time of the row where the fifth star is located is determined and the first attitude update is completed. After that, based on the attitude information determined by the row of the fifth star, we predict the $K\left(t_{2}|t_{1}\right)$ at the row time of the 10th star, and correct Stars 6–9 to the exposure time of the 10th star. The corrected innovation $\Delta K\left(t_{2}\right)$ is fused with the recursive $K\left(t_{2}|t_{1}\right)$ matrix to obtain $K\left(t_{2}|t_{2}\right)$. Hence, the attitude information at the exposure time of the row where the 10th star is located is determined and the second attitude update is completed. According to this method, a single-frame star map can achieve three attitude measurements at different moments.

**Figure 6:**
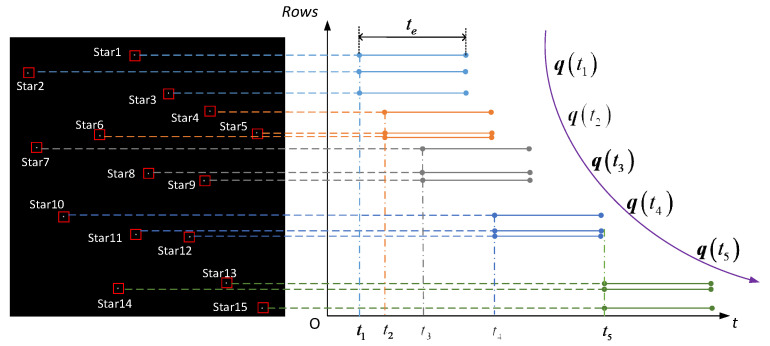
Schematic diagram of updating the attitude every three star points in a single-frame star map.

**Figure 7:**
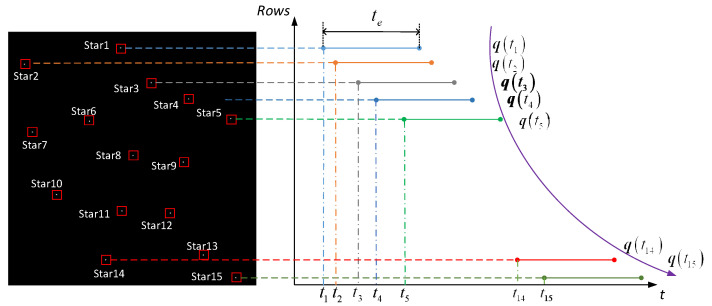
Schematic diagram of updating the attitude for every star point in a single-frame star map.

Similarly, if the attitude is updated once every three stars, five attitude measurements can be achieved in a single frame of the star map, as shown in Figure 6. In the extreme case, the attitude is updated every single star point and each single star point represents a moment. At this time, there is no need for the centroid correction process of the star point, and up to 15 attitude measurements can be achieved in a single frame star map, as shown in Figure 7. Therefore, in a single frame image, the number of attitude updates can be selected according to the number of star points, and the attitude update rate is much higher than that of using a traditional single-frame star map to determine an attitude once. When the number of star point centroid extractions is large, the algorithm can select every star point to update the attitude according to the specific actual update rate requirements. In the actual space environment, various factors such as stray light, noise and electromagnetic radiation can cause interference. Even if the number of star points proposed by the star point centroid extraction algorithm is small, the algorithm can still be suitable for this special case. The detailed steps of the proposed algorithm are described in Algorithm 1.

**Algorithm 1.** High-Update-Rate Attitude Measurement of Star Sensors.**  Input:**  The initial attitude, $q_{0}$; the initial time, $t_{1}$; the initial feature matrix, $K\left(t_{1}\right)$; the initial angular velocity, $\omega \left(t_{1}\right)$; the centroid coordinates of the star point i at the time of the current frame $t_{1}\sim t_{2}$, $\left(x_{i},y_{i}\right)$; the speed of the star point i at the current frame $t_{j}\sim t_{j+1}$ time, $v_{xi}$ and $v_{yi}$; attitude update times, *M*.1: **begin**2: **for**
*j* = 1:*M* − 1 **do**3:  $\Psi \left(t_{j+1},t_{j}\right)\leftarrow \frac{\mathrm{Sin}\left(||\omega \left(t_{j}\right)||\left(t_{j+1}-t_{j}\right)/2\right)}{\left||\omega \left(t_{j}\right)|\right|}\omega \left(t_{j}\right)$4:  $\Phi \left[t_{j+1},\omega \left(t_{j}\right)\right]\leftarrow \mathrm{coS}\left(\frac{1}{2}||\omega \left(t_{j}\right)||\left(t_{j+1}-t_{j}\right)\right)I_{4}+\left[\begin{array}{cc} -\left[\Psi \left(t_{j+1},t_{j}\right)\times \right] & \Psi \left(t_{j+1},t_{j}\right)\\ -\Psi {\left(t_{j+1},t_{j}\right)}^{T} & 0 \end{array}\right]$5:  $K\left(t_{j+1}|t_{j}\right)\leftarrow \Phi [t_{j+1},\omega \left(t_{j}\right)]K\left(t_{j}\right)\Phi^{T}[t_{j+1},\omega \left(t_{j}\right)]$6:   **Function** Star point centroid correction $\left(x_{i},y_{i},v_{xi},v_{yi},k_{j}\right)$7:   $\left[\begin{array}{c} x_{Ci}\\ y_{Ci} \end{array}\right]\leftarrow \left[\begin{array}{c} x_{i}\\ y_{i} \end{array}\right]-\left[\begin{array}{c} \left(y_{i}-k_{j}\right)v_{xi}\Delta t\\ \left(y_{i}-k_{j}\right)v_{yi}\Delta t \end{array}\right]$8:   Calculate $\Delta S_{t_{j}\sim t_{j+1}}$, $\Delta \sigma_{t_{j}\sim t_{j+1}}$, $\Delta z_{t_{j}\sim t_{j+1}}$, $\Delta \sigma_{t_{j}\sim t_{j+1}}$ values using all $x_{Ci}$, $y_{Ci}$ during $t_{j}\sim t_{j+1}$9:   $\Delta K\left(t_{j+1}\right)\leftarrow \left[\begin{array}{cc} \Delta S_{t_{j}\sim t_{j+1}}-\Delta \sigma_{t_{j}\sim t_{j+1}}I & \Delta z_{t_{j}\sim t_{j+1}}\\ \Delta z_{t_{j}\sim t_{j+1}}{}^{T} & \Delta \sigma_{t_{j}\sim t_{j+1}} \end{array}\right]$10:   **end**11:  $K\left(t_{j+1}|t_{j+1}\right)\leftarrow \frac{b_{t_{j}}}{b_{t_{j+1}}}K\left(t_{j+1}|t_{j}\right)+\frac{1}{b_{t_{j+1}}}\Delta K\left(t_{j+1}\right)$12:  Calculate $K\left(t_{j+1}|t_{j+1}\right)$ to obtain $q\left(t_{j+1}|t_{j+1}\right)$13:  **end**
14: **End**

## 4. Experiment and Analysis

### 4.1. Simulation Experiment Analysis

In the simulation environment, the system error was deemed to be corrected. The detector noise was regarded as Gaussian distributed noise and was added to the simulated star map [29]. In this study, the rolling shutter exposure star map was simulated according to the row of the global exposure star point, the speed of the star point’s movement and the exposure delay time between the lines. In total, 3000 Magnitude 6 stars were selected from the SAO catalog [30] to construct a star database to ensure that the navigation stars entered and left the field of view under dynamic conditions. Table 1 lists the main parameters of the star sensor. The simulation experiment analysis was conducted from the following three aspects: (1) attitude angle error analysis at different update rates at the same angular velocity, (2) attitude angle mean root square error analysis at different angular velocities and different update rates, and (3) attitude update time analysis and comparisons. When the angular velocity was 0.05 degrees/s, 300 image frames were continuously sampled, and 15 star points in each frame were selected as the navigation stars. The star points were evenly distributed on the star map in the initial frame. Table 2 lists the position coordinates of the star points.

**Figure 8:**
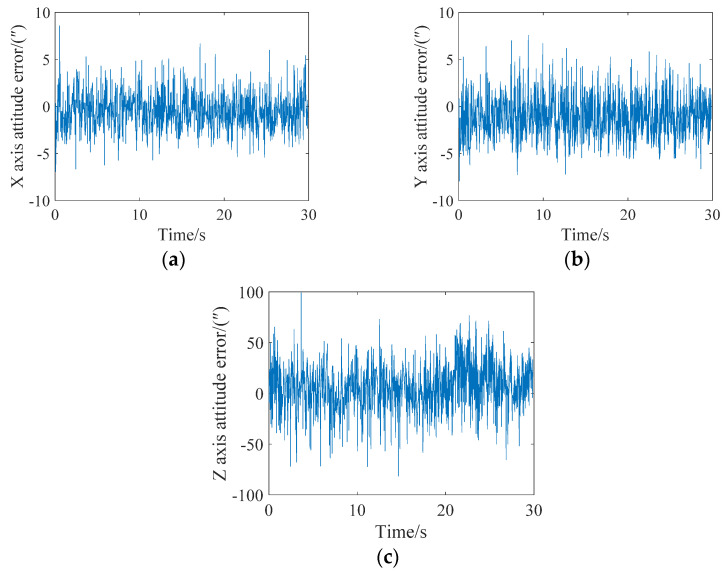
Attitude error analysis when the angular velocity is 0.05 degrees/s and the attitude is updated every five stars. (**a**) X-axis; (**b**) Y-axis; (**c**) Z-axis.

After every fifth star point had been corrected to the same time, an attitude update was completed, that is, Star 1–Star 5 corrected the target row with the behavior of Star 5, Star 6–Star 10 corrected the target row with the behavior of Star 10, and so on. The attitude angle error analysis of the final three axes (X, Y and Z) is shown in Figure 8. It can be seen from the figure that the maximum errors of the X, Y and Z axes are less than 9″, 9″ and 100″, respectively. The attitude update could be achieved three times in the image.

**Figure 9:**
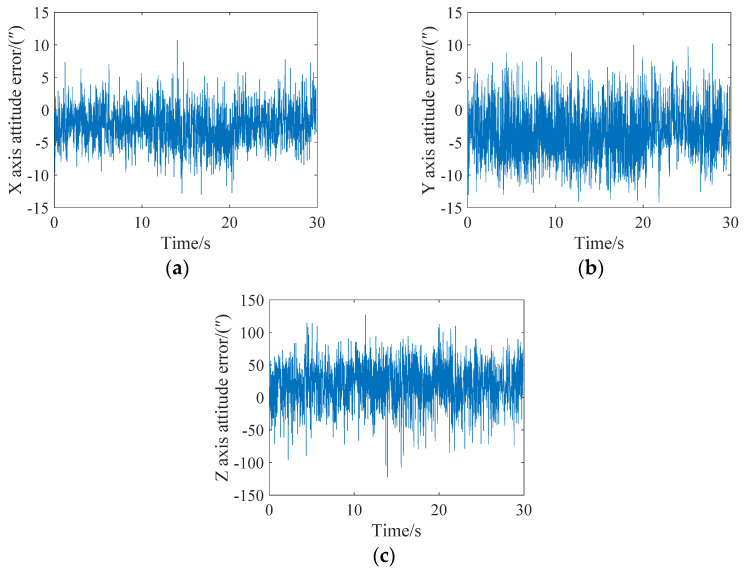
Attitude error analysis when the angular velocity is 0.05 degrees/s and the attitude is updated every three stars. (**a**) X-axis; (**b**) Y-axis; (**c**) Z-axis.

Next, the attitude update was completed every three star points, that is, Star 1–Star 3 corrected the target row with the behavior of Star 3, Star 4–Star 6 corrected the target row with the behavior of Star 6, and so on. The attitude angle error analysis of the X, Y, and Z axes is shown in Figure 9. It can be seen from the figure that the maximum errors of the X, Y and Z axes are less than 10″, 15″, and 150″, respectively. Thus, a single-frame star map realized five posture updates.

**Figure 10:**
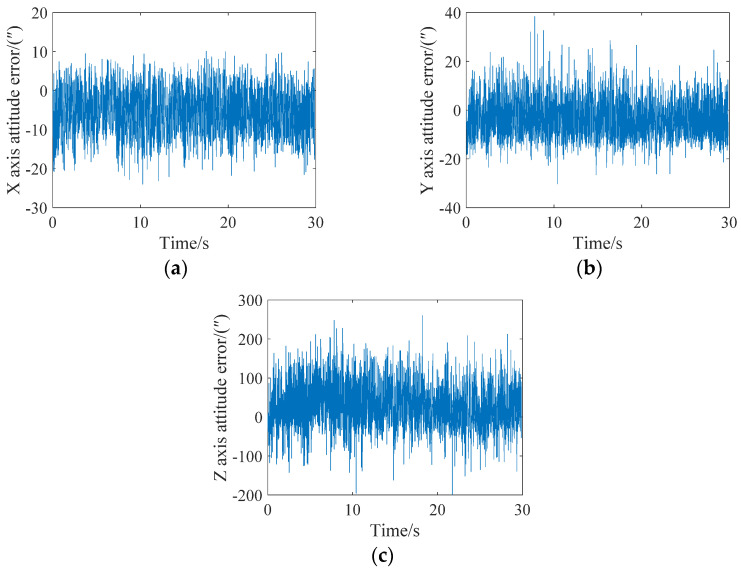
Attitude error analysis when the angular velocity is 0.05 degrees/s and the attitude is updated every star. (**a**) X-axis; (**b**) Y-axis; (**c**) Z-axis.

In extreme cases, an attitude update can be completed for every star point. The attitude angle error analysis of the X, Y and Z axes is shown in Figure 10. It can be seen from the figure that the maximum errors of the X, Y and Z axes is less than 15″, 30″ and 200″, respectively. A single frame of the star map can achieve 15 attitude updates.

The root mean square errors of the attitude errors in the three different situations are compared in Table 3. It can be seen from the table that the more star points involved in the correction to the same time, the smaller the attitude error. The main reason is that the more star points, the more effective attitude information in the matrix, and the more accurate the posture calculation.

**Figure 11:**
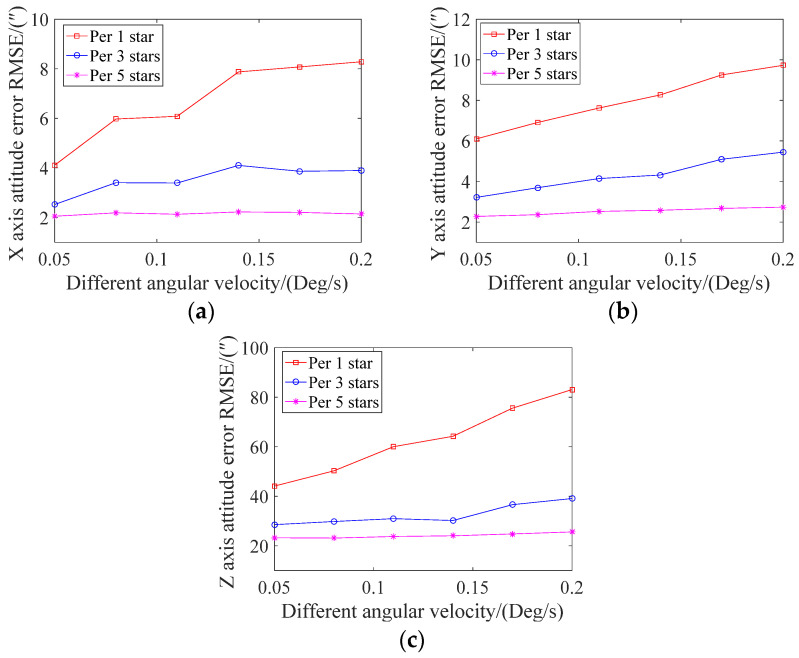
The mean square errors of the attitude angle in three different situations at different angular velocities. (**a**) X-axis; (**b**) Y-axis; (**c**) Z-axis.

The attitude error under different angular velocities is shown in Figure 11. It can be seen from the figure that the X, Y, and Z axes’ errors for each star point and all three updates of the attitude increased with the increase in angular velocity. Moreover, the attitude error was relatively stable when the attitude was updated once every five star points. At 0.2 degrees/s, the X and Y axes’ attitude angle errors were all within 10″ in all three cases, while the Z-axis had a relatively large error. When each star point was updated, the error exceeded 80″.

**Figure 12:**
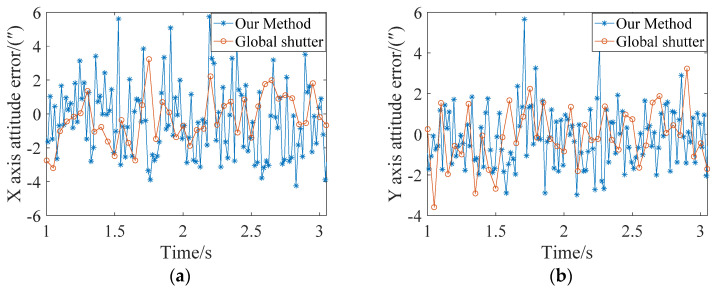

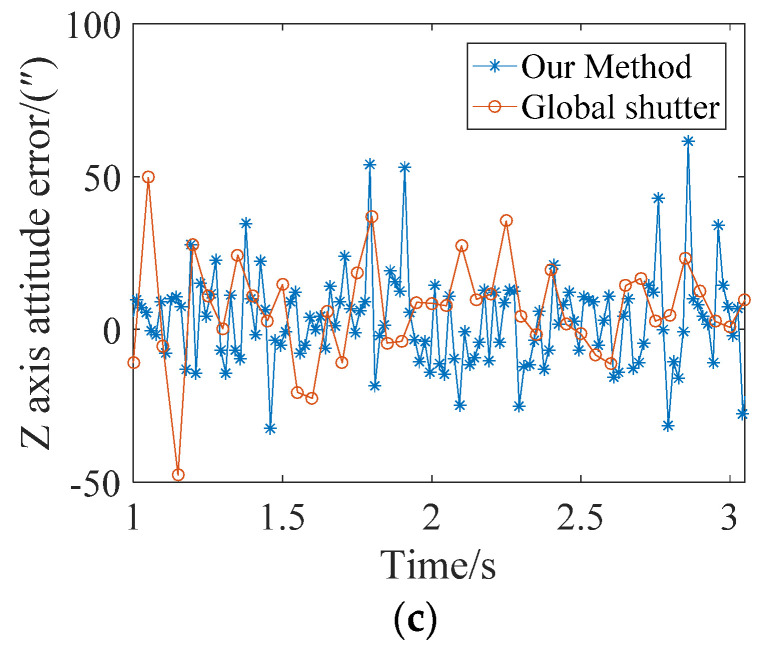
Comparison of attitude errors between our method and global exposure. (**a**) X-axis; (**b**) Y-axis; (**c**) Z-axis.

Figure 12 compares the attitude errors of rolling shutter exposure with every five stars being corrected and global shutter exposure using 15 stars to calculate the attitude. It can be seen from the figure that the attitude accuracy of the global exposure on the X, Y, and Z axes is slightly higher than that of the rolling shutter exposure after correction, mainly because there are 15 star points involved in the calculation of each frame of the image during the global exposure. However, at the same time, since the posture can only be determined once in a single frame of the global exposure, when the posture was updated every five stars in the rolling exposure, the posture update rate was three times that of the global exposure.

**Figure 13:**
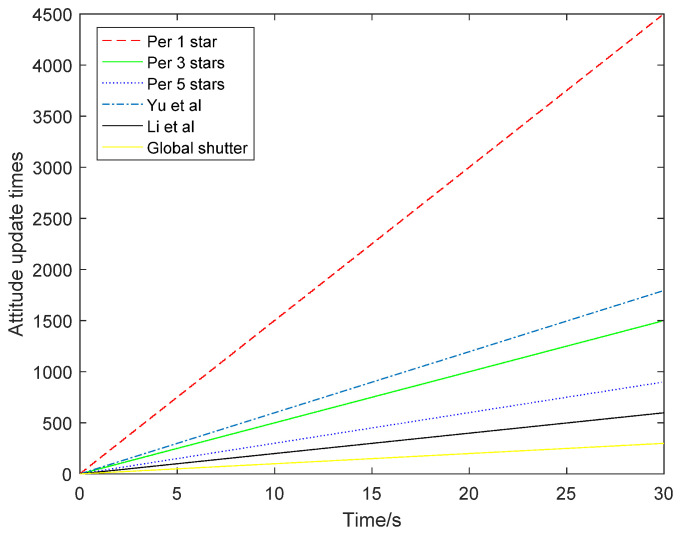
Comparison of attitude update times.

The attitude update times of the three different situations are compared in Figure 13. When the attitude was updated once for each star point, the update times were the highest, reaching 4500 times, and the attitude update rate was 150 Hz. However, the accuracy of its attitude angle was relatively low. When the attitude was updated every five stars, the attitude accuracy was higher but the attitude update rate was only 30 Hz. When the attitude was updated every three stars, the attitude update rate was 50 Hz and the accuracy was between the former two. Therefore, the choice of attitude update rate and accuracy can be determined according to specific actual requirements.

Compared with the other methods, when the attitude was updated once per star point, the number of updates was the same as that of the method based on the extended Kalman filter. However, the method proposed in this article can flexibly choose the attitude update rate according to the needs of different scenarios. The posture update rate was increased by 2.5 times compared with the method proposed by Yu Wenbo et al., and is 7.5 times higher than the method proposed by Li Xinpeng et al. When the attitude was updated every three stars, it was 2.5 times higher than the method proposed by Li Xinpeng and others. When the attitude was updated every five stars, the attitude update rate was 1.5 times higher than that of the other methods, including the method proposed by Li Xinpeng. Moreover, since the methods proposed by Yu Wenbo et al. and Li Xinpeng et al. all improved the attitude update rate via the hardware approach, which would increase power consumption and volume, this article can solve this problem from the perspective of algorithms. 

### 4.2. Experiment

**Figure 14:**
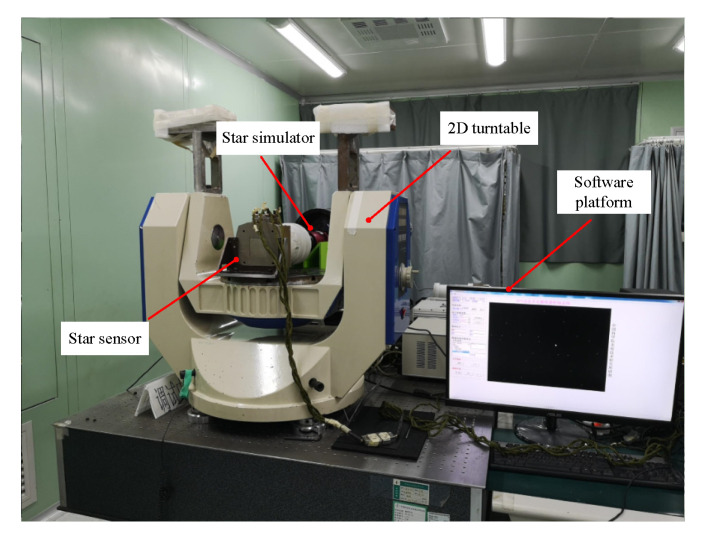
Experimental platform.

The experiment used the Star1000 star sensor, the static star simulator and the high-precision 2D turntable independently developed by the Institute of Optoelectronics of the Chinese Academy of Sciences. The specific parameters of the Star1000 star sensor are listed in Table 4. The star sensor was installed and fixed in the center of the turntable. The static star simulator was placed in front of the operating table and the star sensor lens was aimed at the static star model. Figure 14 shows the experimental setup. Through rotation of the turntable, the environment of the real star sensor in the orbit was simulated. At the same time, the software platform was used to collect the navigation star point information taken by the star sensor.

In the experiment, there were eight effective navigation stars captured by the star sensor. According to the distribution of star points in each frame of the star map, the attitude calculation was performed every four stars, every two stars and every single star using the high-update-rate attitude measurement algorithm of the star sensor based on the rolling shutter exposure. In a single-frame star map, two, four and eight attitude updates could be achieved, respectively.

**Figure 15:**
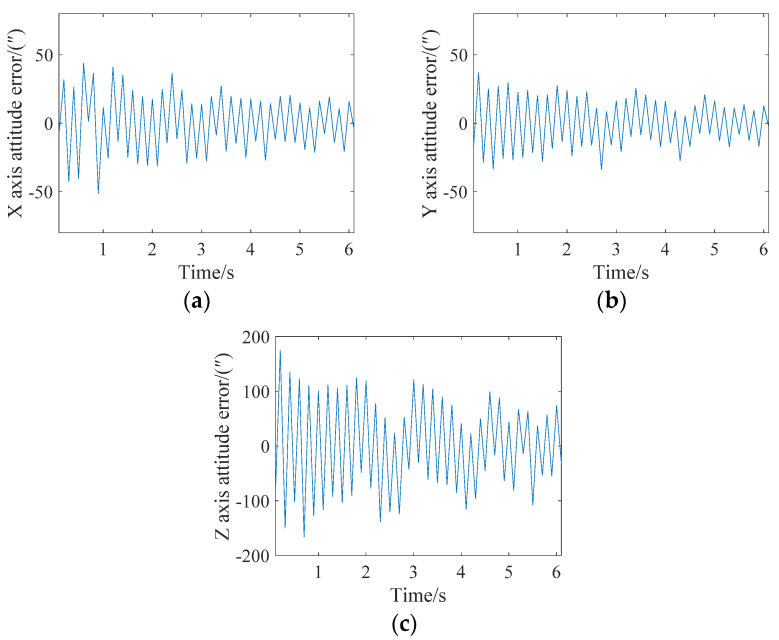
The attitude error updated every four stars at 0.1 degree/s. (**a**) X-axis; (**b**) Y-axis; (**c**) Z-axis.

**Figure 16:**
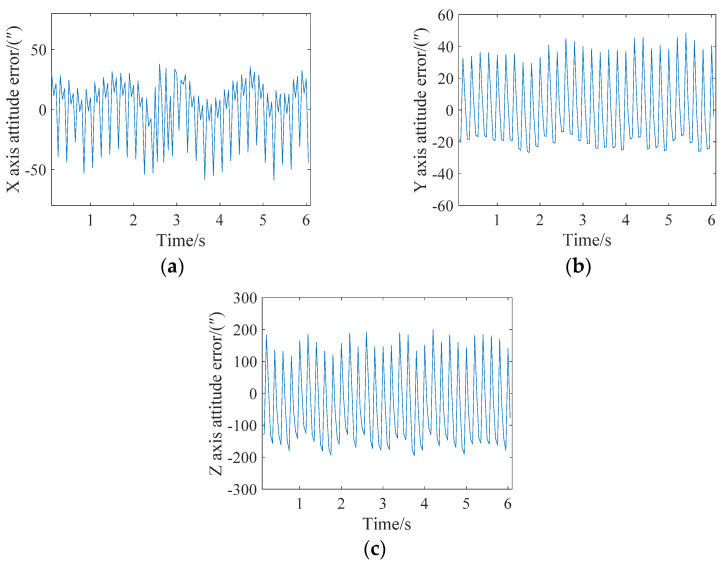
The attitude error updated every two stars at 0.1 degrees/s. (**a**) X-axis; (**b**) Y-axis; (**c**) Z-axis.

**Figure 17:**
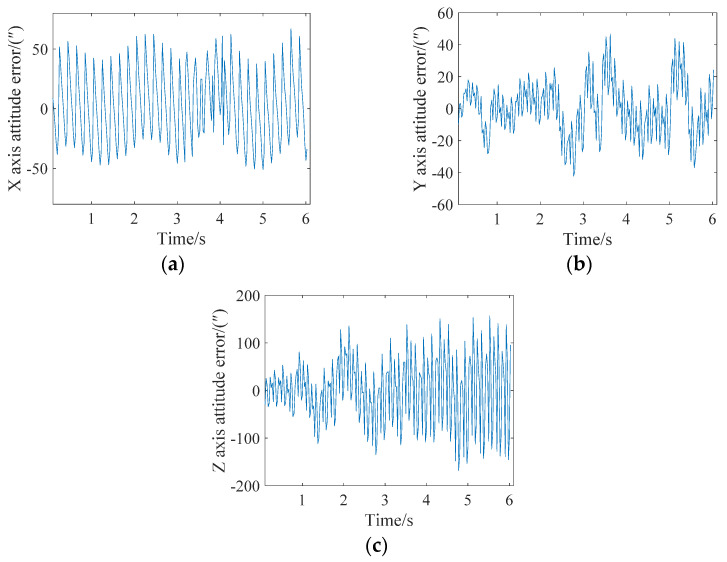
The attitude error updated every star at 0.1 degrees/s. (**a**) X-axis; (**b**) Y-axis; (**c**) Z-axis.

The attitude errors of updating the attitude every four stars, every two stars and every single star when the angular velocity was 0.1 degrees/s are shown in Figure 15, Figure 16 and Figure 17, respectively. It can be seen from the comparison that the accuracy of the X and Y axes was higher than that of the Z-axis in the three different situations. Among these, when the attitude was updated every four stars, the maximum error of the attitude angles of the X and Y axes was less than 40″ and 30″, respectively. When the attitude was updated every two stars, the maximum error of the attitude angles of the X and Y axes was less than 50″ and 40″, respectively. When the attitude was updated every single star, the maximum error of the attitude angles of the X and Y axes was less than 60″ and 50″, respectively. When the number of star points during calibration is large, the accuracy of the attitude angle will be relatively high because the matrix formed by more star points will have more feature information for determining the attitude.

At the same time, when the attitude angle accuracy was higher, the corresponding attitude update times were lower. Because the exposure time was 100 ms, the time between the frames of the rolling shutter exposure was about 200 ms. Within 6 s, the attitude was updated once every four stars in a single frame, and there were two attitude information points in one frame and a total of 60 attitude information points. Similarly, after every second star point was corrected, the attitude information was updated once and there was a total of 120 attitude information points. When each star point was updated once, there was a total of 240 attitude information points. In Figure 15, Figure 16 and Figure 17, the error curves are denser.

**Figure 18:**
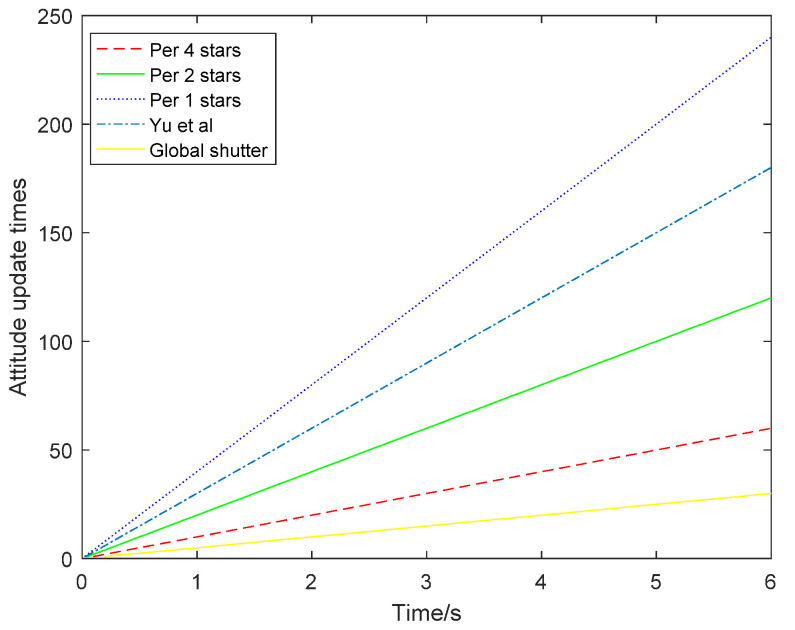
Comparison of attitude update times within 6 s.

In Figure 18, compared with the traditional global exposure, the attitude was updated once per frame of the star map, and the attitude update rate increased by two times, four times and eight times, from the original 5 Hz to 10 Hz, 20 Hz and 40 Hz, respectively. The update times of the attitude update per star point are the same as the update times of the method based on the extended Kalman filter [8], which is 1.3 times higher than the attitude update rate of the method proposed by Yu Wenbo et al. [5]. Compared with the method proposed by Li Xinpeng et al. [6], the attitude update rate is four times higher than that of updating the attitude every single star point. The attitude update rate is twice that of updating the attitude every two stars and the attitude update rate is the same as that of updating attitude every four stars. Moreover, the method proposed in this study can be adapted to the requirements of different scenarios, and the attitude update rate of the star sensor can be selected according to the specific number and distribution of star points.

## 5. Conclusions

In order to solve the problem of the low attitude update rate of a star sensor, a method with a high attitude update rate based on star point correction of rolling shutter exposure is proposed in this study. From the perspective of the software algorithm, the proposed method is based on the pose information contained in the asynchronous rolling shutter exposure method, and the star sensor is recursively estimated with the corrected star point information in the star map. The posture is updated many times. The proposed method can select the attitude update rate of the star sensor based on the number and distribution of the star points in the star map. Moreover, the proposed method can be adapted to the requirements of different attitude update rates in space missions, which provides an effective solution for breaking through the technical barriers of the low attitude update rate of traditional star sensors.

## Figures and Tables

**Table 1:** The main parameters of the star sensor.

Parameter	Value
Focal length	24.09 mm
Field of view	10° × 10°
Image size	1024 × 1024
Pixel size	5.5 um
Exposure time	50 ms
Delay time between rows	49 us

**Table 2:** The position coordinates of the 15 initial stars in the detector.

Star	Coordinates (um)	Star	Coordinates (um)
1	(1006.90, 126.67)	9	(247.78, 654.28)
2	(745.58, 183.83)	10	(183.59, 674.60)
3	(666.62, 306.83)	11	(721.39, 768.83)
4	(944.63, 316.96)	12	(880.11, 780.64)
5	(658.98, 332.85)	13	(513.59, 802.82)
6	(114.30, 562.38)	14	(449.36, 936.26)
7	(881.07, 591.60)	15	(112.47, 991.47)
8	(554.01, 620.24)		

**Table 3:** Comparison of root mean square errors of the attitude errors in different situations.

	X-axis (RMSE)	Y-axis (RMSE)	Z-axis (RMSE)
Per 1 star	4.1096	6.1013	44.1262
Per 3 stars	2.5250	3.2098	28.5692
Per 5 stars	2.0503	2.2750	23.2379

**Table 4:** The parameters of the Star1000 star sensor.

Parameter	Value
Focal length	43.279 mm
Field of view	20° × 20°
Image size	1024 × 1024
Pixel size	15 μm
Exposure time	100 ms
Delay time between rows	97.7 μs
